# Transcriptome analysis reveals a reprogramming energy metabolism-related signature to improve prognosis in colon cancer

**DOI:** 10.7717/peerj.9458

**Published:** 2020-07-07

**Authors:** Xinxin Zhang, Jinyuan Xu, Yujia Lan, Fenghua Guo, Yun Xiao, Yixue Li, Xia Li

**Affiliations:** 1 College of Bioinformatics Science and Technology, Harbin Medical University, Harbin, Heilongjiang, China; 2 Key Laboratory of Cardiovascular Medicine Research, Harbin Medical University, Harbin, China

**Keywords:** Colon cancer, Overall survival, Reprogramming energy metabolism, Signature, Metabolism

## Abstract

Although much progress has been made to improve treatment, colon cancer remains a leading cause of cancer death worldwide. Metabolic reprogramming is a significant ability of cancer cells to ensure the necessary energy supply in uncontrolled proliferation. Since reprogramming energy metabolism has emerged as a new hallmark of cancer cells, accumulating evidences have suggested that metabolism-related genes may serve as key regulators of tumorigenesis and potential biomarkers. In this study, we analyzed a set of reprogramming energy metabolism-related genes by transcriptome analysis in colon cancer and revealed a five-gene signature that could significantly predict the overall survival. The reprogramming energy metabolism-related signature could distinguish patients into high-risk and low-risk groups with significantly different survival times (*P* = 0.0011; HR = 1.92; 95% CI [1.29–2.87]). Its prognostic value was confirmed in another two independent colon cancer cohorts (*P* = 5.2e–04; HR = 2.09, 95%; CI [1.37–3.2] for GSE17538 and *P* = 3.8e−04; HR = 2.08, 95% CI [1.37–3.16] for GSE41258). By multivariable analysis, we found that the signature was independent of clinicopathological features. Its power in promoting risk stratification of the current clinical stage was then evaluated by stratified analysis. Moreover, the signature could improve the power of the TNM stage for the prediction of overall survival and could be used in patients who received adjuvant chemotherapy. Overall, our results demonstrated the important role of the reprogramming energy metabolism-related signature in promoting stratification of high-risk patients, which could be diagnostic of adjuvant therapy benefit.

## Introduction

Colon cancer is the third most common cause of cancer-related death in the United States and one of the most prevalent diseases worldwide (Bray et al., 2018; Siegel, Miller & Jemal, 2019). In the past decades, many studies devoted to understanding the tumorigenesis of colon cancer to descend its mortality. Unfortunately, the prognosis of colon cancer remains pessimistic for many patients. In clinical trials, pathological staging is the primary prognostic classification to select patients for adjuvant chemotherapy. The tumor–node–metastasis (TNM) staging system has been confirmed as a conventional classification system (Pineros et al., 2019). Depending on the TNM stage at the time of diagnosis, a proper possibility of curative surgical intervention and clinical survival time can be predicted (Boland & Goel, 2016; Vychytilova-Faltejskova et al., 2016). However, the TNM staging fails to predict recurrence accurately in many patients undergoing curative surgery and the prediction is feasible mainly for patients limited to the primary tumor and regional lymph nodes. For the patients with the same stage and receive similar treatment, they may have distinct clinical outcomes (Das, Kalita & Pal, 2017).

Metabolic reprogramming confers cancer cells with the ability to alter cellular metabolism to support the increased energy request due to continuous growth, rapid proliferation, and other characteristics (Cazzaniga & Bonanni, 2015). It is therefore not surprising that altered cellular metabolism in cancer cells is critical to meet the anabolic demands of cancer cells (Claudino et al., 2012; Hanahan & Weinberg, 2011). Importantly, reprogramming energy metabolism has emerged as a hallmark of cancer and is required for malignant cell development, including invasion and metastasis (Hanahan & Weinberg, 2011). Current efforts have proved that metabolic reprogramming has been pointed out as a promising target for anti-cancer therapy (Granja et al., 2015). Exploration of energy metabolism heterogeneity has been proved to be beneficial to discover the useful biomarkers for cancers (Li, Zhan & Zhan, 2018). Many studies have implicated cancer metabolism as a major contributor to tumor initiation, growth and metastatic dissemination in colorectal cancer (La Vecchia & Sebastian, 2020). The alteration of specific metabolic pathways, including nucleotide metabolism, amino acid metabolism and carbohydrate metabolism, could influence the carcinogenesis in colorectal cancer (Manna et al., 2014; Xie et al., 2015). Some of the driver genes of colorectal cancer also play critical roles in controlling metabolic reprogramming, such as *KRAS* and *p53* (Kawada, Toda & Sakai, 2017; Labuschagne, Zani & Vousden, 2018). Although the interest in the metabolic reprogramming of cancer cells is arising in the past few years, few studies focus on the expression and clinical significance of the metabolism-related genes in colon cancer. Therefore, it would be valuable to explore a reprogramming energy metabolism-related signature for prognosis and risk stratification for improving the management of colon cancer patients.

Here, based on multiple independent cohorts of colon cancer, we evaluated the expression and prognostic value of metabolism-related genes and analyzed a reprogramming energy metabolism-related signature that would be able to predict the overall survival of patients. The results indicated that the signature could serve as an independent prognostic factor and promote the risk stratification according to TNM stages for the prediction of overall survival.

## Materials and Methods

### Colon cancer datasets preparation

Three independent and publicly available gene expression microarray datasets (GSE39582 (Marisa et al., 2013), GSE17538 (Freeman et al., 2012; Smith et al., 2010; Williams et al., 2015a), and GSE41258 (Martin et al., 2018; Sheffer et al., 2009)), which also contained patient outcome and clinicopathological features, were retrieved from the Gene Expression Omnibus (GEO). In total, 879 colon cancer patients were included. For the dataset GSE39582, we excluded 86 samples which received treatment but not without corresponding drug information or did not contain the exact treatment information. The 316 patients who didn’t receive adjuvant chemotherapy and the 19 normal subjects from GSE39582 were treated as training set, and the other two datasets (GSE17538 and GSE41258) were served as independent validation cohorts. In the training cohort, 3 tumor samples that had no available clinical survival information and the 19 normal samples were excluded in survival analysis. For the two validation cohorts, we used the similar strategies to select samples. Besides, 164 colon cancer patients who received fluorouracil-based ACT in GSE39582 were further used for stratification analysis.

### Identification of reprogramming energy metabolism-related genes associated with prognosis in colon cancer

Differential expression analyses to detect transcriptome differences between tumor and normal samples were performed by the “limma” R package (3.42.2) in GSE39582. We assigned the false discovery rate (FDR) as 0.1% and fold change (FC) threshold as 2 to screen differentially expressed genes (DEGs). Among DEGs, we focused on genes involved in reprogramming energy metabolism as well as genes related to malignant tissue development. The reprogramming energy metabolism-related genes were identified by hallmark-associated GO terms in our previous research (Deng et al., 2018). Then, we performed the univariable Cox regression analysis to select genes associated with overall survival in the training cohort. Finally, a risk score model was established: Risk score = (0.4911 × expression level of *AARS*) + (0.2992 × expression level of *COL4A2*) + (0.2865 × expression level of *COL4A1*) + (−0.3543 × expression level of *PCNA*) + (−0.2377 × expression level of *MMP12*). The formula was a linear combination of the five genes weighted by its corresponding regression coefficient in the univariable Cox regression analysis. Based on this model, each sample could be assigned an assessment score and then assigned to the high-risk or low-risk group according to the median score in the corresponding cohort.

### Statistical analysis

The Kaplan–Meier method was used to assess the survival time in each cohort (Dudley, Wickham & Coombs, 2016). The difference of overall survival between the high-risk group and the low-risk group in each cohort was evaluated by the log-rank test. We applied the univariate Cox regression model to assess the prognostic value of the signature and performed the multivariate Cox regression models to estimate whether it was an independent predictor of overall survival in colon cancer (Zhang et al., 2016b). The Cox proportional hazards regression model was used to calculated hazard ratio (HR) and 95% confidence intervals (CI). The statistically significant result was defined as the two-tailed *P*-value being less than 0.05. We further performed ROC analysis to assess the sensitivity and specificity of the signature in overall survival prediction. The prediction models of the five-gene risk score and the TNM stage were also assessed, respectively. And then, the two variables were regarded as predictors and combined in a logistic regression model. The pROC package was used to plot ROC curves of the predictions (Robin et al., 2011), and the areas under the curve (AUC) of these models were computed to compare their abilities. All the statistical analyses were conducted under the R program 3.5.2 (http://www.r-project.org).

## Result

### Transcriptome analysis reveals a prognostic signature related to reprogramming energy metabolism

Transcriptional profiles of colon cancer patients and corresponding clinical information were obtained from Marisa et al. (2013) After removing patients who received fluorouracil-based ACT or without available clinical survival information, a total of 316 tumor samples and 19 normal samples were recruited in the training cohort (Table 1). Based on the transcriptional profile, we identified 1,489 differentially expressed genes in colon cancer patients (FDR < 0.001 and FC > 2 by “limma” method), including 752 up-regulated and 737 down-regulated genes. Then, by subjecting the differentially expressed genes to reprogramming energy metabolism as well as malignant tissue development, we obtained 43 metabolism-related genes for subsequent analysis (Table S1). To extract a core prognostic gene set related to reprogramming energy metabolism, we applied univariate Cox proportional hazards regression analysis. We obtained a five-gene signature that was significantly related to overall survival (Table 2, *P* < 0.05). Among the five genes, high expression levels of two genes (*PCNA* and *MMP12*) were strongly associated with better overall survival, and the other three genes (*COL4A1*, *COL4A2*, and *AARS*) showed opposite correlations. Based on the risk score formula of the five genes (see Materials and Methods), each sample assigned a risk score (Fig. S1). Then, samples could be divided into high-risk (*n* = 156) and low-risk (*n* = 157) groups according to the median score 3.71 in the training cohort in which 3 tumor samples without available clinical survival information were excluded. For the high-risk group, *COL4A1*, *COL4A2*, and *AARS* showed relatively higher expression and *PCNA* and *MMP12* showed relatively lower expression than the low-risk group. Moreover, the high-risk samples presented a trend of short survival time (Fig. S2).

**Table 1:** The Clinical characteristics of patients in the three independent cohorts.

**Characteristic**	**GSE39582(*N* = 499)**	**GSE17538(*N* = 232)**	**GSE41258(*N* = 167)**
**Age at diagnosis, years**	67.8	65.5	66.0
**Gender**			
Male	275	122	87
Female	224	110	80
**Sample Type**			
Tumor	480	232	167
Normal	19	–	–
**TNM Stage of tumor**			
Stage 0	4	–	–
I	33	28	26
II	248	72	39
III	158	76	43
IV	37	56	59
**Chemotherapy of tumor**			
NO	316	–	–
YES	164	–	–

**Notes.**

TNM Tumor node metastasis

### Validation of the prognostic value of the reprogramming energy metabolism-related signature

**Figure 1:**
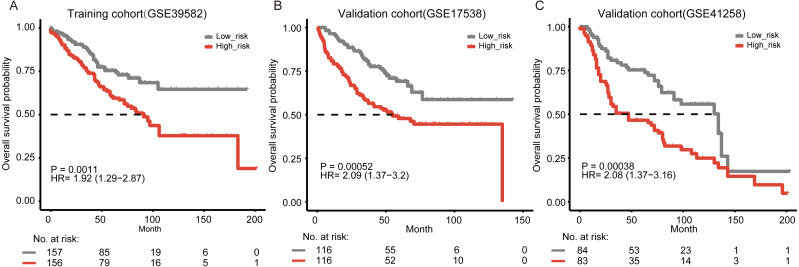
Kaplan–Meier curves of overall survival according to the five-gene signature. (A) Training cohort (GSE39582, *n* = 313). In the training cohort, three tumor samples that had no available clinical survival information and 19 normal samples were removed in survival analysis. (B) Validation cohort from GSE17538 (*n* = 232). (C) Validation cohort from GSE41258 (*n* = 167). The two-side log-rank test determined the differences between the two curves.

To evaluate the prognostic value of the signature, we performed Kaplan–Meier analysis to evaluate the survival outcomes of patients with colon cancer. Our result showed that the overall survival time of the patients in the high-risk group was significantly shorter than that in the low-risk group (Fig. 1A, log-rank test, *P* = 0.0011). By using univariate Cox regression analysis, we also found that patients with higher five-gene risk scores had shorter overall survival time (hazard ratio (HR) = 1.92, 95% CI [1.29–2.87]).

**Table 2:** List of the 5 genes significantly correlated with the overall survival in the training cohort.

Gene symbol	*P*-value	HR	Coefficient
AARS	0.041^*^	1.634	0.4911
COL4A2	0.035^*^	1.349	0.2992
COL4A1	0.037^*^	1.332	0.2865
PCNA	0.032^*^	0.702	−0.3543
MMP12	<0.001^*^	0.788	−0.2377

**Notes.**

* Significant *P* values are labeled with (*P* < 0.05).

To further validate the prognostic ability of the five-gene signature, we collected another two independent validation cohorts, i.e., GSE17538 (*n* = 232) and GSE41258 (*n* = 167). Consistent with the results in the training cohort, patients classified as “high-risk” suffered significantly poorer overall survival than those defined as “low-risk” in both two independent validation cohorts (Figs. 1B–1C, *P* = 5.2e−04 and 3.8e−04, respectively). Through the univariate Cox regression model analysis, the correlation between the signature and the overall survival was consistently strong in the two cohorts (HR = 2.09, 95% CI [1.37–3.2] for GSE17538 and HR = 2.08, 95% CI [1.37–3.16] for GSE41258). In addition, we collected 373 colon cancer samples containing RNA-seq expression and clinical information from The Cancer Genome Atlas (TCGA) (https://cancergenome.nih.gov/). Our signature was still able to significantly predict overall survival of colon cancer patients in TCGA (Fig. S3A, *P* = 0.038, HR = 1.57, 95% CI [1.02–2.4]). We further explored our risk model in other gastro-intestinal tumors from TCGA but not of colonic origin. We found that the risk model did not have the ability to predict patient survival in other gastro-intestinal tumors (Figs. S3B–S3C, *P* = 0.8, HR = 0.85, 95% CI [0.23–3.17] for pancreatic cancer and *P* = 0.096, HR = 0.69, 95% CI [0.45–1.07] for liver cancer). Our signature might be specific for colon tissue.

### The five-gene signature predicts patient survival independently

To further test whether the prognostic value of the five-gene signature was independent, we performed the multivariate Cox regression model analysis according to clinicopathological parameters of the colon cancer patients, including age, sex, and tumor stage. Our results proved that the signature remained to have prognostic power of overall survival when considering those factors in the training cohort (Table 3, *P* = 0.009, HR = 1.72, 95% CI [1.15–2.59]). The multivariate Cox regression model analysis based on the two validation cohorts also showed that the risk score was significant among all clinicopathological parameters and significantly associated with overall survival (Table 3, *P* = 0.006, HR = 1.84, 95% CI [1.19–2.82] for GSE17538 and *P* = 0.01, HR = 1.76, 95% CI [1.14–2.71] for GSE41258, respectively). While for other factors, only the TNM stage was independent in all cohorts (Table 3). In addition, we further explored the connection between the signature and the TNM stages. The risk scores had a positive Spearman’s correlation with the TNM stages (Fig. S4, Spearman’s correlation, 0.26 for the training cohort; 0.24 for the validation cohort GSE17538; 0.271 for the validation cohort GSE41258).

**Table 3:** Univariate and multivariate Cox regression analyses in the three colon cohorts.

**Variables**		**Univariate analysis**			**Multivariate analysis**		
		HR	95% CI of HR	*P*-value	HR	95% CI of HR	*P*-value
**Training set GSE39582**							
Risk score							
	High-risk VS Low-risk	1.956	1.306–2.931	0.001^*^	1.722	1.146–2.588	0.009^*^
Age		1.045	1.026–1.065	<0.001^*^	1.046	1.025–1.067	<0.001^*^
Gender							
	Male VS Female	1.460	0.983–2.169	0.061	2.015	1.331–3.052	0.001^*^
TNM Stage							
	III-IV VS I-II	2.559	1.714–3.820	<0.001^*^	2.310	1.526–3.497	<0.001^*^
**Validation set GSE17538**							
Risk score							
	High-risk VS Low-risk	2.090	1.366–3.199	0.001^*^	1.836	1.194–2.823	0.006^*^
Age		1.009	0.992–1.025	0.304	1.020	1.004–1.037	0.016^*^
Gender							
	Male VS Female	1.006	0.669–1.515	0.975	1.105	0.720–1.694	0.648
TNM Stage							
	III-IV VS I-II	3.696	2.230–6.127	<0.001^*^	3.775	2.256–6.319	<0.001^*^
**Validation set GSE41258**							
Risk score							
	High-risk VS Low-risk	2.082	1.372–3.161	0.001^*^	1.760	1.144–2.708	0.010^*^
Age		1.007	0.991–1.024	0.370	1.014	0.996–1.031	0.122
Gender							
	Male VS Female	1.659	1.082–2.542	0.020^*^	1.748	1.126–2.715	0.013^*^
TNM Stage							
	III-IV VS I-II	3.878	2.395–6.280	<0.001^*^	3.668	2.238–6.012	<0.001^*^

**Notes.**

TNM Tumor node metastasis

* Significant *P* values are labeled with (*P* < 0.05).

### Prognostic prediction of the signature within clinical stages

**Figure 2:**
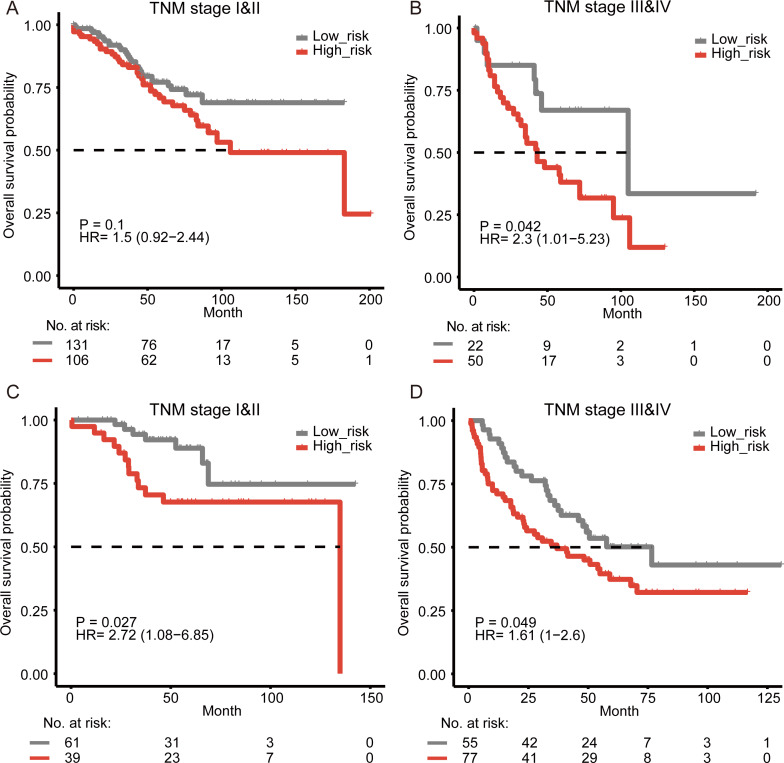
Survival analysis for colon cancer patients stratified by the TNM stage. The Kaplan–Meier estimates overall survival in stage I–II patients and stages III–IV patients from the training cohort (A–B) and the validation cohort from GSE17538 (C–D).

The disease-specific survival of colon cancer is impacted by several factors, including the tumor–node–metastasis (TNM) staging. The stage of disease at diagnosis has been proved to be a major predictor to help evaluate the prognosis (Crooke et al., 2018). In the multivariate survival analysis, our results showed that the prognostic value of the signature was significant in all cohorts and independent of TNM stage. To examine the significance of our reprogramming energy metabolism-related signature in patients with the same stage, a stratified analysis was performed. Our results showed that the signature could further divide the subgroups of patients with the same stage. Those patients with high-grade (Stage III–IV) tumors were divided into two subgroups with significantly different survival times (Fig. 2B, log-rank test, *P* = 0.042). For patients with low-stage (Stage I–II) tumors, our signature could also divide them into two different prognostic groups (Fig. 2A, log-rank test, *P* = 0.1). Similar results were also found in the other two cohorts (Figs. 2C–2D, Fig. S5).

**Figure 3:**
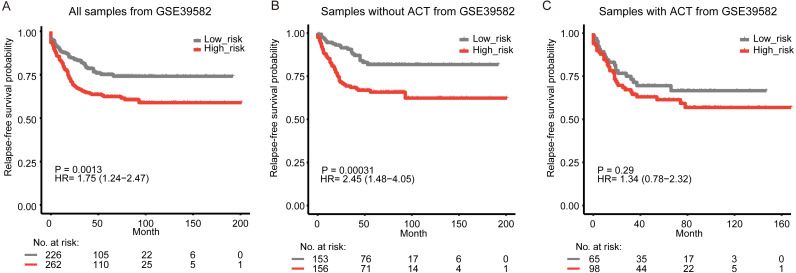
Kaplan-Meier curves of relapse-free survival according to the signature in GSE39582. All samples with and without fluorouracil-based adjuvant chemotherapy (ACT) (A), the samples without ACT (B), and the samples without ACT (C).

### The signature is associated with adjuvant chemotherapy and can improve the predictive value of TNM stage

**Figure 4:**
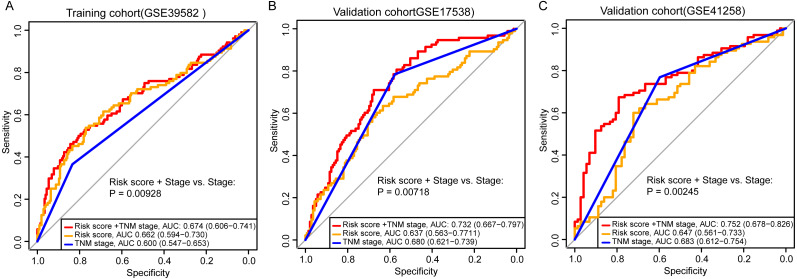
Comparison of sensitivity and specificity for survival prediction by the signature and TNM stage. The ROC curves evaluating the prediction capability of the five-gene risk score, the TNM stage, and the combination of the two factors in GSE39582 (A), GSE17538 (B), and GSE41258 (C). *P*-value showed the AUC of the TNM stage versus the AUC of the combination of signature and TNM stage.

Adjuvant chemotherapy is frequently considered for patients with colon cancer. We found that the signature could significantly predict overall survival and relapse-free survival for all samples with and without fluorouracil-based adjuvant chemotherapy (ACT) in GSE39582 (Fig. S6A, Fig. 3A, log-rank test, *P* = 0.0013 for overall survival; *P* = 0.0013 for relapse-free survival). The high-risk samples who did not receive ACT, had a significantly worse overall survival and were more likely to relapse (Figs. 1A, 3B, log-rank test, *P* = 0.0011 for overall survival; *P* = 0.00031 for relapse-free survival). For the samples who received ACT, there was a similar phenomenon, but without significance (Fig. S6B, Fig. 3C, log-rank test, *P* = 0.14 for overall survival; *P* = 0.29 for relapse-free survival). The results implied that the samples could benefit from adjuvant chemotherapy without a significant difference in risk scores. To assess the combinative prognostic value of the signature with clinical factors, we further analyzed the benefit of the signature by evaluating the risk score together with the TNM stage. As shown in Table 3, the risk score and the TNM stage were both independent prognostic factors. So, it is reasonable to generate a new score combining the risk score and the TNM stage. When considering the reprogramming energy metabolism-related signature together with TNM stage, the prognostic power of TNM stage could be significantly enhanced: the AUC was 0.60 (95% CI [0.55–0.65]) for TNM stage alone, and the AUC became 0.67 (95% CI [0.61–0.74]) when adding the risk score (Fig. 4A, *P* = 0.0093). The other two independent cohorts also showed significant improvement after adding the risk score (*P* = 0.0072 for GSE17538 and *P* = 0.0025 for GSE41258, Figs. 4B and 4C).

## Discussion

In this study, we found the prognostic power of metabolism-related genes and identified a reprogramming energy metabolism-related signature to predict overall patient survival in colon cancer. The prognosis value of the signature was robust among three independent cohorts in both Kaplan–Meier and univariate analyses. After adjusting clinicopathological risk factors, this signature could serve as an independent prognostic factor and improve the classification based on the TNM stage. In addition, we also observed that the signature could predict the survival benefits of adjuvant chemotherapy for colon cancer patients.

Metabolic reprogramming, which is required for both malignant transformation and tumor development, has been recognized as one of the hallmarks of tumor cells. Accumulating evidences have revealed that metabolic reprogramming could remarkably enhance the invasion and metastatic potentials of cancer cells and can facilitate the development of novel therapeutic strategies (Yoshida, 2015). Therefore, our reprogramming energy metabolism-related signature could be helpful to explore the importance of metabolic reprogramming and develop innovative therapeutic strategy. The advances in metabolic researches of colonic epithelium and serum have provided the possibility to identify individuals who may have potential risk for the development of colon cancer and could explored guidance on the ongoing management of patients (Williams et al., 2015b). The dysregulation of some genes in metabolism, such as fatty acid metabolism and lipid metabolism, had an important role in tumor progression and prognosis of human cancers (Nath & Chan, 2016; Sun et al., 2015). For example, Chen et al. screened two differentially expressed metabolic genes, *PGK1* and *G6PD*, which were determined as critical regulators involved in glycolysis and the pentose phosphate pathway, respectively. Their up-regulated expression is associated with a high risk of recurrent metastasis and poor survival of patients (Chen et al., 2018; Chen et al., 2020). Moreover, antimetabolites have been used in many modern chemotherapy regimens that improve patient survival and, in some cases, help cure disease (Luengo, Gui & Van der Heiden, 2017). Thus, understanding the molecular features in tumor metabolism could reveal potential value in better interpreting the progression of colon cancer.

For the five genes in the signature, four of them (expected *COL4A1*) were annotated in the metabolic process (GO:0008152). *PCNA* was one of the members in the metabolism of proteins pathway (PathCards, https://pathcards.genecards.org/) and played an essential role in nucleic acid metabolism (Kelman, 1997). Deletion of *MMP12* could effectively prevent inducible *NOS* and then contribute to the glucose metabolism (Bahadoran, Mirmiran & Ghasemi, 2020). Using proteomic analysis, Chafey et al. identified *AARS* was involved in amino-acid metabolism pathway (Chafey et al., 2009). Both *COL4A1* and *COL4A2* were involved in the PI3K-Akt pathway and this signaling pathway has been demonstrated to be responsible for controlling metabolic reprogramming (Lien, Lyssiotis & Cantley, 2016; Liu et al., 2020). All of five genes have been previously reported to have prognostic roles in several cancers, such as *AARS* in breast cancer and *PCNA* in colon adenocarcinoma (Ho et al., 2017; Klupp et al., 2016; Zhang et al., 2016a). Matrix metalloproteinases (MMPs) are important in colorectal cancer invasion and progression. Among them, *MMP12* is known to be associated with increased survival in colorectal cancer, which has become an important therapeutic target (Said, Raufman & Xie, 2014). Previous studies have shown that *COL4A1* is essential in tumorigenesis, which contributes to the proliferation, migration and colony formation in many cancers (Huang et al., 2018; Jin et al., 2017). *COL4A1* has been identified as one of the prognostic biomarkers of several diseases, whose high expression level is usually associated with poor overall survival (consistent with our result HR = 1.332) (Huang et al., 2018; Li et al., 2019; Sulpice et al., 2013). Another marker, *COL4A2*, is involved in proliferation and migration of endothelial cells and induces apoptosis (JingSong et al., 2017). A correlation analysis of *COL4A2* showed that the overexpression of *COL4A2* was highly correlated with shorter progression-free survival in liver cancer (Liu et al., 2020). Heterotrimers composed of *COL4A1* and *COL4A2* constituted the most abundant components of almost all basement membranes. It has been found that *COL4A1/2* could accelerate cell cycle and promote tumorigenesis (Liu et al., 2020). Kuo et al. proved that mutations in *COL4A1* or *COL4A2* were important to a broad spectrum of disorders and may be potential therapeutic targets (Kuo, Labelle-Dumais & Gould, 2012). In addition, it has been reported that *COL4A1* and *COL4A2* were associated with high risk of relapse in colon cancer (Chida et al., 2016). They were predominantly expressed in the cancer stroma and were further found to be specifically expressed by the endothelial cells and cancer-associated fibroblasts. A recent study showed that cancer cell metabolism had been proposed to drive stromal cells toward a regenerative response that supports tumor growth (Schworer, Vardhana & Thompson, 2019). Considering the mutant phenotypes of both *COL4A1* and *COL4A2* included metabolism phenotype and the GO annotations related to them contained extracellular matrix structural constituent (Eppig, 2017), the two metabolism-related genes may be released by tumor cells and mainly play a role in cancer stroma. The fluorescence-based real-time polymerase chain reaction (qPCR) has become the gold standard for the quantification of mRNAs and ensconced as the benchmark for clinical prognosis and pathogen detection (Bustin, 2010). We will further validate our signature with potential biomarker value by qPCR to expand the clinical application.

## Conclusions

In conclusion, our study presented a powerful reprogramming energy metabolism-related signature for the prognosis of colon cancer by employing different independent patient cohorts. The risk score was significantly separate among other clinicopathological factors and could be used as a potential clinical predictor alongside the TNM stage. The five-gene signature would be useful to develop novel strategies of precision medicine, and more prospective studies are necessary to further assess the reliability and the clinical implications of the reprogramming energy metabolism-related signature.

##  Supplemental Information

10.7717/peerj.9458/supp-1 Supplemental Information 1 Supplemental Figures

10.7717/peerj.9458/supp-2 Supplemental Information 2 The list of 43 differentially expressed metabolism-related genes

## Additional Information and Declarations

### Data Availability

The following information was supplied regarding data availability: Data is available at NCBI GEO: GSE39582, GSE17538, GSE41258.

## References

[ref-1] Bahadoran Z, Mirmiran P, Ghasemi A (2020). Role of nitric oxide in insulin secretion and glucose metabolism. Trends in Endocrinology and Metabolism.

[ref-2] Boland CR, Goel A (2016). Prognostic subgroups among patients with stage II colon cancer. New England Journal of Medicine.

[ref-3] Bray F, Ferlay J, Soerjomataram I, Siegel RL, Torre LA, Jemal A (2018). Global cancer statistics 2018: GLOBOCAN estimates of incidence and mortality worldwide for 36 cancers in 185 countries. CA: A Cancer Journal for Clinicians.

[ref-4] Bustin SA (2010). Why the need for qPCR publication guidelines?–The case for MIQE. Methods.

[ref-5] Cazzaniga M, Bonanni B (2015). Relationship between metabolic reprogramming and mitochondrial activity in cancer cells. Understanding the anticancer effect of metformin and its clinical implications. Anticancer Research.

[ref-6] Chafey P, Finzi L, Boisgard R, Cauzac M, Clary G, Broussard C, Pegorier JP, Guillonneau F, Mayeux P, Camoin L, Tavitian B, Colnot S, Perret C (2009). Proteomic analysis of beta-catenin activation in mouse liver by DIGE analysis identifies glucose metabolism as a new target of the Wnt pathway. Proteomics.

[ref-7] Chen J, Cao S, Situ B, Zhong J, Hu Y, Li S, Huang J, Xu J, Wu S, Lin J, Zhao Q, Cai Z, Zheng L, Wang Q (2018). Metabolic reprogramming-based characterization of circulating tumor cells in prostate cancer. Journal of Experimental & Clinical Cancer Research.

[ref-8] Chen J, Ye C, Dong J, Cao S, Hu Y, Situ B, Xi X, Qin S, Xu J, Cai Z, Zheng L, Wang Q (2020). Metabolic classification of circulating tumor cells as a biomarker for metastasis and prognosis in breast cancer. Journal of Translational Medicine.

[ref-9] Chida S, Okayama H, Noda M, Saito K, Nakajima T, Aoto K, Hayase S, Momma T, Ohki S, Kono K, Takenoshita S (2016). Stromal VCAN expression as a potential prognostic biomarker for disease recurrence in stage II-III colon cancer. Carcinogenesis.

[ref-10] Claudino WM, Goncalves PH, Leo Adi, Philip PA, Sarkar FH (2012). Metabolomics in cancer: a bench-to-bedside intersection. Critical Reviews in Oncology/Hematology.

[ref-11] Crooke H, Kobayashi M, Mitchell B, Nwokeji E, Laurie M, Kamble S, McKenna M, Masood A, Korytowsky B (2018). Estimating 1- and 5-year relative survival trends in colorectal cancer (CRC) in the United States: 2004 to 2014. Journal of Clinical Oncology.

[ref-12] Das V, Kalita J, Pal M (2017). Predictive and prognostic biomarkers in colorectal cancer: a systematic review of recent advances and challenges. Biomedicine and Pharmacotherapy.

[ref-13] Deng Y, Luo S, Zhang X, Zou C, Yuan H, Liao G, Xu L, Deng C, Lan Y, Zhao T, Gao X, Xiao Y, Li X (2018). A pan-cancer atlas of cancer hallmark-associated candidate driver lncRNAs. Molecular Oncology.

[ref-14] Dudley WN, Wickham R, Coombs N (2016). An introduction to survival statistics: Kaplan–Meier Analysis. Journal of the Advanced Practitioner in Oncology.

[ref-15] Eppig JT (2017). Mouse Genome Informatics (MGI) Resource: genetic, genomic, and biological knowledgebase for the laboratory mouse. Institute for Laboratory Animal Research Journal.

[ref-16] Freeman TJ, Smith JJ, Chen X, Washington MK, Roland JT, Means AL, Eschrich SA, Yeatman TJ, Deane NG, Beauchamp RD (2012). Smad4-mediated signaling inhibits intestinal neoplasia by inhibiting expression of beta-catenin. Gastroenterology.

[ref-17] Granja S, Pinheiro C, Manuel Reis R, Martinho O, Baltazar F (2015). Glucose addiction in cancer therapy: advances and drawbacks. Current Drug Metabolism.

[ref-18] Hanahan D, Weinberg RA (2011). Hallmarks of cancer: the next generation. Cell.

[ref-19] Ho YJ, Lin YM, Huang YC, Shi B, Yeh KT, Gong Z, Lu JW (2017). Prognostic significance of high YY1AP1 and PCNA expression in colon adenocarcinoma. Biochemical and Biophysical Research Communications.

[ref-20] Huang R, Gu W, Sun B, Gao L (2018). Identification of COL4A1 as a potential gene conferring trastuzumab resistance in gastric cancer based on bioinformatics analysis. Molecular Medicine Reports.

[ref-21] Jin R, Shen J, Zhang T, Liu Q, Liao C, Ma H, Li S, Yu Z (2017). The highly expressed COL4A1 genes contributes to the proliferation and migration of the invasive ductal carcinomas. Oncotarget.

[ref-22] JingSong H, Hong G, Yang J, Duo Z, Li F, WeiCai C, XueYing L, YouSheng M, YiWen O, Yue P, Zou C (2017). siRNA-mediated suppression of collagen type iv alpha 2 (COL4A2) mRNA inhibits triple-negative breast cancer cell proliferation and migration. Oncotarget.

[ref-23] Kawada K, Toda K, Sakai Y (2017). Targeting metabolic reprogramming in KRAS-driven cancers. International Journal of Clinical Oncology.

[ref-24] Kelman Z (1997). PCNA: structure, functions and interactions. Oncogene.

[ref-25] Klupp F, Neumann L, Kahlert C, Diers J, Halama N, Franz C, Schmidt T, Koch M, Weitz J, Schneider M (2016). Serum MMP7, MMP10 and MMP12 level as negative prognostic markers in colon cancer patients. BMC Cancer.

[ref-26] Kuo DS, Labelle-Dumais C, Gould DB (2012). COL4A1 and COL4A2 mutations and disease: insights into pathogenic mechanisms and potential therapeutic targets. Human Molecular Genetics.

[ref-27] La Vecchia S, Sebastian C (2020). Metabolic pathways regulating colorectal cancer initiation and progression. Seminars in Cell and Developmental Biology.

[ref-28] Labuschagne CF, Zani F, Vousden KH (2018). Control of metabolism by p53 - Cancer and beyond. Biochimica et Biophysica Acta - Reviews on Cancer.

[ref-29] Li DF, Wang NN, Chang X, Wang SL, Wang LS, Yao J, Li ZS, Bai Y (2019). Bioinformatics analysis suggests that COL4A1 may play an important role in gastric carcinoma recurrence. Journal of Digestive Diseases.

[ref-30] Li N, Zhan X, Zhan X (2018). Energy metabolism heterogeneity-based molecular biomarkers for ovarian cancer. Molecular Medicine: IntechOpen.

[ref-31] Lien EC, Lyssiotis CA, Cantley LC (2016). Metabolic reprogramming by the PI3K-Akt-mTOR pathway in cancer. Recent Results in Cancer Research.

[ref-32] Liu Y, Zhang J, Chen Y, Sohel H, Ke X, Chen J, Li YX (2020). The correlation and role analysis of COL4A1 and COL4A2 in hepatocarcinogenesis. Aging.

[ref-33] Luengo A, Gui DY, Van der Heiden MG (2017). Targeting metabolism for cancer therapy. Cell Chemical Biology.

[ref-34] Manna SK, Tanaka N, Krausz KW, Haznadar M, Xue X, Matsubara T, Bowman ED, Fearon ER, Harris CC, Shah YM, Gonzalez FJ (2014). Biomarkers of coordinate metabolic reprogramming in colorectal tumors in mice and humans. Gastroenterology.

[ref-35] Marisa L, De Reynies A, Duval A, Selves J, Gaub MP, Vescovo L, Etienne-Grimaldi MC, Schiappa R, Guenot D, Ayadi M, Kirzin S, Chazal M, Flejou JF, Benchimol D, Berger A, Lagarde A, Pencreach E, Piard F, Elias D, Parc Y, Olschwang S, Milano G, Laurent-Puig P, Boige V (2013). Gene expression classification of colon cancer into molecular subtypes: characterization, validation, and prognostic value. PLOS Medicine.

[ref-36] Martin ML, Zeng Z, Adileh M, Jacobo A, Li C, Vakiani E, Hua G, Zhang L, Haimovitz-Friedman A, Fuks Z, Kolesnick R, Paty PB (2018). Logarithmic expansion of LGR5(+) cells in human colorectal cancer. Cellular Signalling.

[ref-37] Nath A, Chan C (2016). Genetic alterations in fatty acid transport and metabolism genes are associated with metastatic progression and poor prognosis of human cancers. Scientific Reports.

[ref-38] Pineros M, Parkin DM, Ward K, Chokunonga E, Ervik M, Farrugia H, Gospodarowicz M, O’Sullivan B, Soerjomataram I, Swaminathan R, Znaor A, Bray F, Brierley J (2019). Essential TNM: a registry tool to reduce gaps in cancer staging information. The Lancet Oncology.

[ref-39] Robin X, Turck N, Hainard A, Tiberti N, Lisacek F, Sanchez JC, Muller M (2011). pROC: an open-source package for R and S+ to analyze and compare ROC curves. BMC Bioinformatics.

[ref-40] Said AH, Raufman JP, Xie G (2014). The role of matrix metalloproteinases in colorectal cancer. Cancers.

[ref-41] Schworer S, Vardhana SA, Thompson CB (2019). Cancer metabolism drives a stromal regenerative response. Cell Metabolism.

[ref-42] Sheffer M, Bacolod MD, Zuk O, Giardina SF, Pincas H, Barany F, Paty PB, Gerald WL, Notterman DA, Domany E (2009). Association of survival and disease progression with chromosomal instability: a genomic exploration of colorectal cancer. Proceedings of the National Academy of Sciences of the United States of America.

[ref-43] Siegel RL, Miller KD, Jemal A (2019). Cancer statistics, 2019. CA: A Cancer Journal for Clinicians.

[ref-44] Smith JJ, Deane NG, Wu F, Merchant NB, Zhang B, Jiang A, Lu P, Johnson JC, Schmidt C, Bailey CE, Eschrich S, Kis C, Levy S, Washington MK, Heslin MJ, Coffey RJ, Yeatman TJ, Shyr Y, Beauchamp RD (2010). Experimentally derived metastasis gene expression profile predicts recurrence and death in patients with colon cancer. Gastroenterology.

[ref-45] Sulpice L, Rayar M, Desille M, Turlin B, Fautrel A, Boucher E, Llamas-Gutierrez F, Meunier B, Boudjema K, Clément B (2013). Molecular profiling of stroma identifies osteopontin as an independent predictor of poor prognosis in intrahepatic cholangiocarcinoma. Hepatology.

[ref-46] Sun Y, He W, Luo M, Zhou Y, Chang G, Ren W, Wu K, Li X, Shen J, Zhao X, Hu Y (2015). SREBP1 regulates tumorigenesis and prognosis of pancreatic cancer through targeting lipid metabolism. Tumour Biology.

[ref-47] Vychytilova-Faltejskova P, Radova L, Sachlova M, Kosarova Z, Slaba K, Fabian P, Grolich T, Prochazka V, Kala Z, Svoboda M, Kiss I, Vyzula R, Slaby O (2016). Serum-based microRNA signatures in early diagnosis and prognosis prediction of colon cancer. Carcinogenesis.

[ref-48] Williams CS, Bernard JK, Beckler MDemory, Almohazey D, Washington MK, Smith JJ, Frey MR (2015a). ERBB4 is over-expressed in human colon cancer and enhances cellular transformation. Carcinogenesis.

[ref-49] Williams MD, Zhang X, Belton AS, Xian L, Huso T, Park JJ, Siems WF, Gang DR, Resar LM, Reeves R, Hill Jr HH (2015b). HMGA1 drives metabolic reprogramming of intestinal epithelium during hyperproliferation, polyposis, and colorectal carcinogenesis. Journal of Proteome Research.

[ref-50] Xie G, Wang CZ, Yu C, Qiu Y, Wen XD, Zhang CF, Yuan CS, Jia W (2015). Metabonomic profiling reveals cancer chemopreventive effects of american ginseng on colon carcinogenesis in Apc(Min/+) mice. Journal of Proteome Research.

[ref-51] Yoshida GJ (2015). Metabolic reprogramming: the emerging concept and associated therapeutic strategies. Journal of Experimental & Clinical Cancer Research.

[ref-52] Zhang S, Gu Y, Wu S, Kang Y, Liu S, Zhang D (2016a). Identification of differentially expressed genes and prognostic biomarkers of breast cancer based on RNA-Seq and KEGG pathway network. Cancer Genetics and Epigenetics.

[ref-53] Zhang W, Mao JH, Zhu W, Jain AK, Liu K, Brown JB, Karpen GH (2016b). Centromere and kinetochore gene misexpression predicts cancer patient survival and response to radiotherapy and chemotherapy. Nature Communications.

