# Supplementary material for: Transcriptome analysis reveals a reprogramming energy metabolism-related signature to improve prognosis in colon cancer
Source: PeerJ. 2020 Jul 7;8:e9458. doi: 10.7717/peerj.9458 (PMC7350917; doi:10.7717/peerj.9458)
Supplement: Supplemental Information 1 [file peerj-08-9458-s001.pdf]

Supplementary Material

1 Supplementary Figures and Tables

1.1 Supplementary Figures

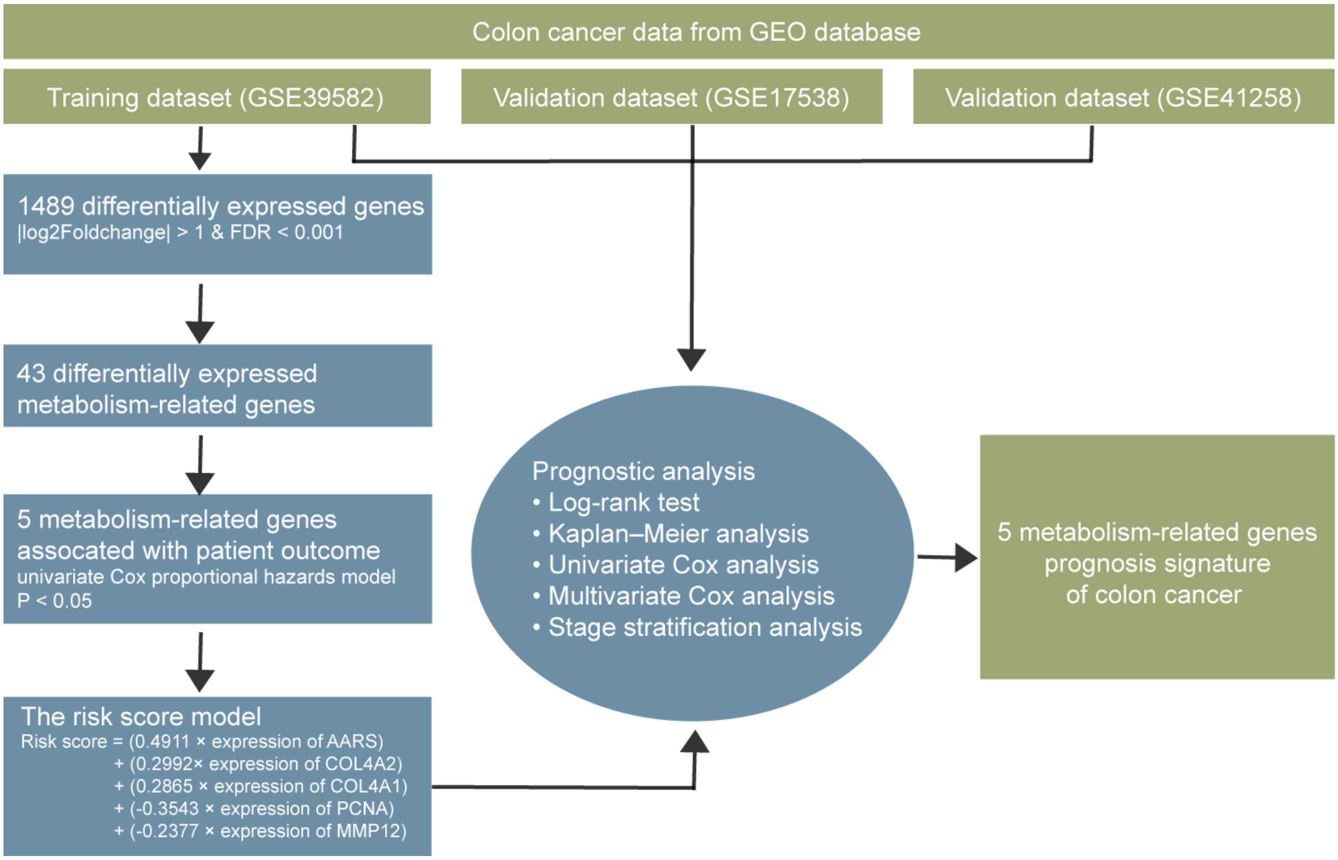

Supplementary Figure 1. Workflow of the identification and validation of the metabolism-related prognosis signature.

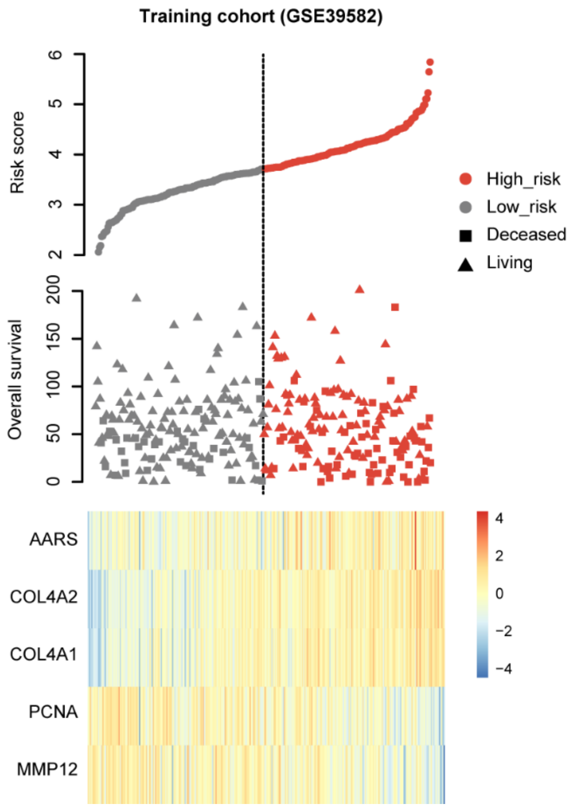

Supplementary Figure 2. Risk score analysis of the training cohort (GSE39582). The distribution of the metabolism-related risk score (Top); patients' overall survival status and time (Middle); heatmap of the expression profiles of the five genes in the signature (Bottom).

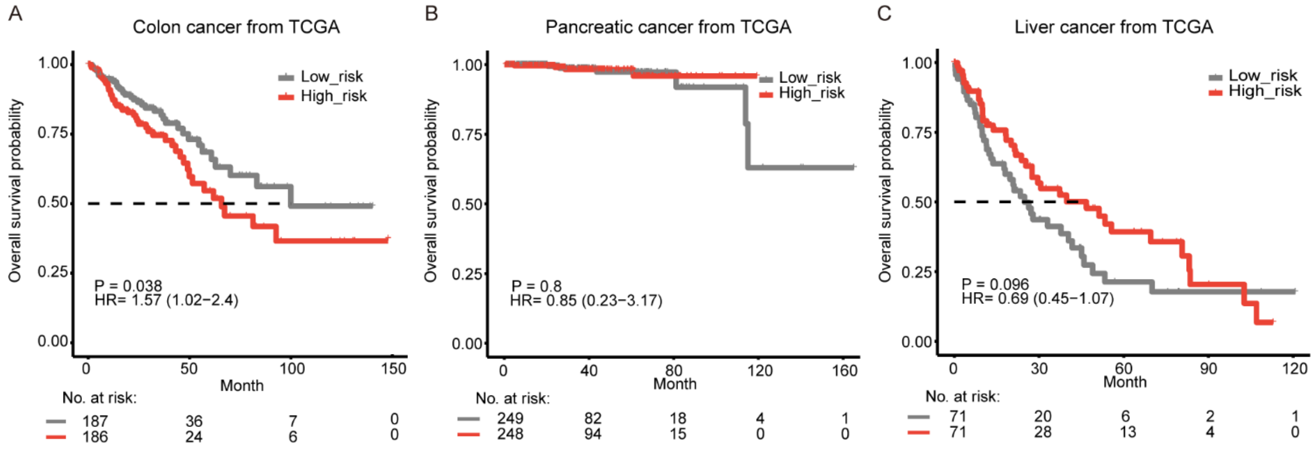

Supplementary Figure 3. Survival analysis for gastro-intestinal tumors from TCGA. (A) Colon cancer. (B) Pancreatic cancer. (C) Liver cancer.

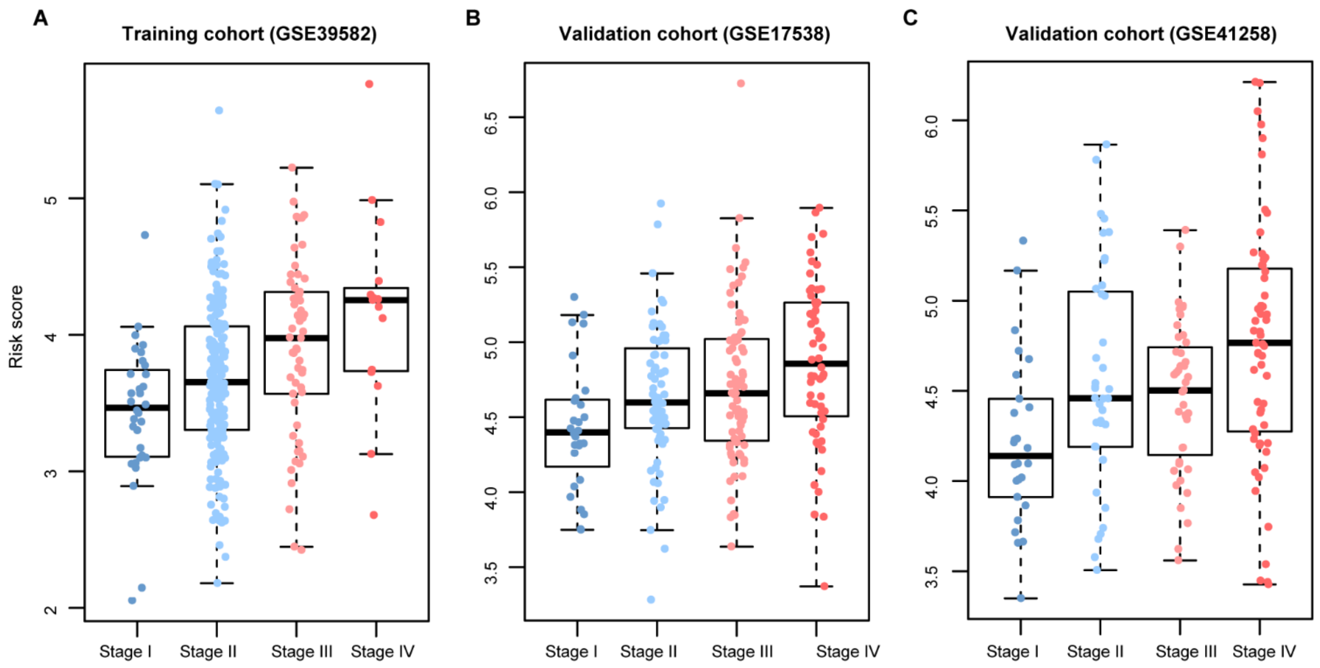

Supplementary Figure 4. Correlation analysis of the risk score and TNM stages. (A) Training cohort from GSE39582. (B) Validation cohort from GSE17538. (C) Validation cohort from GSE41258.

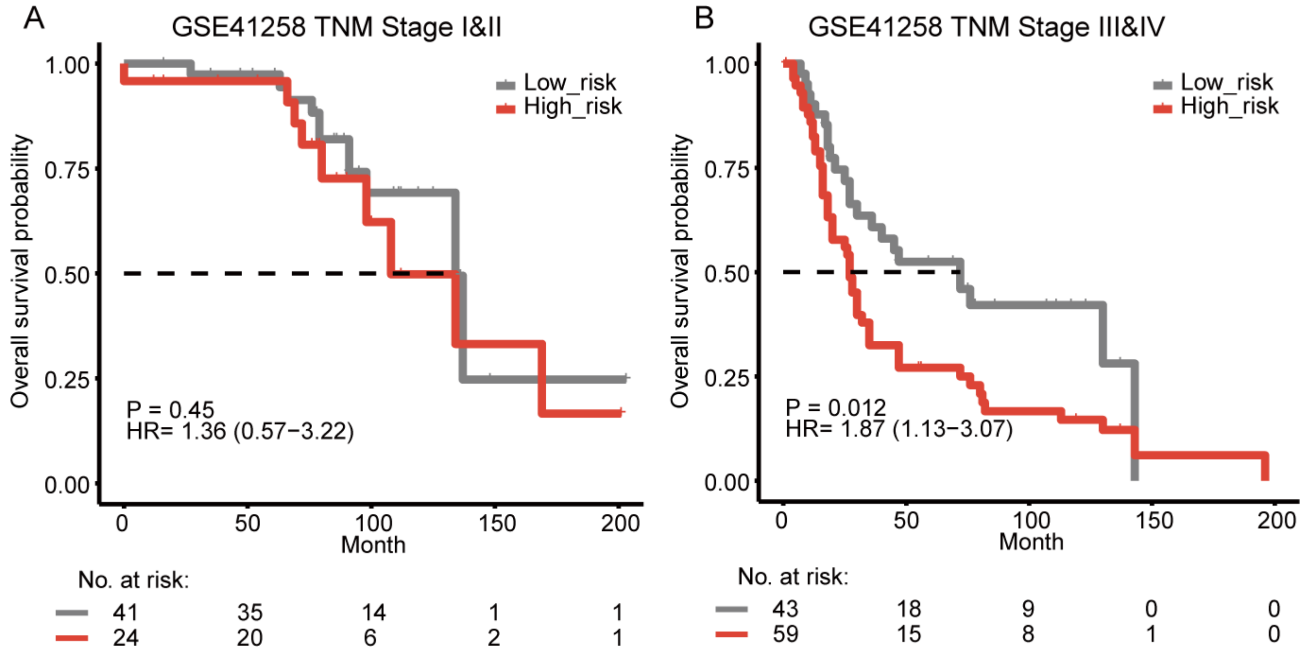

Supplementary Figure 5. Survival analysis for colon cancer patients stratified by TNM stage in GSE41258. The Kaplan–Meier estimates overall survival in stage I-II patients (A) and stage III-IV patients (B) from GSE41258.

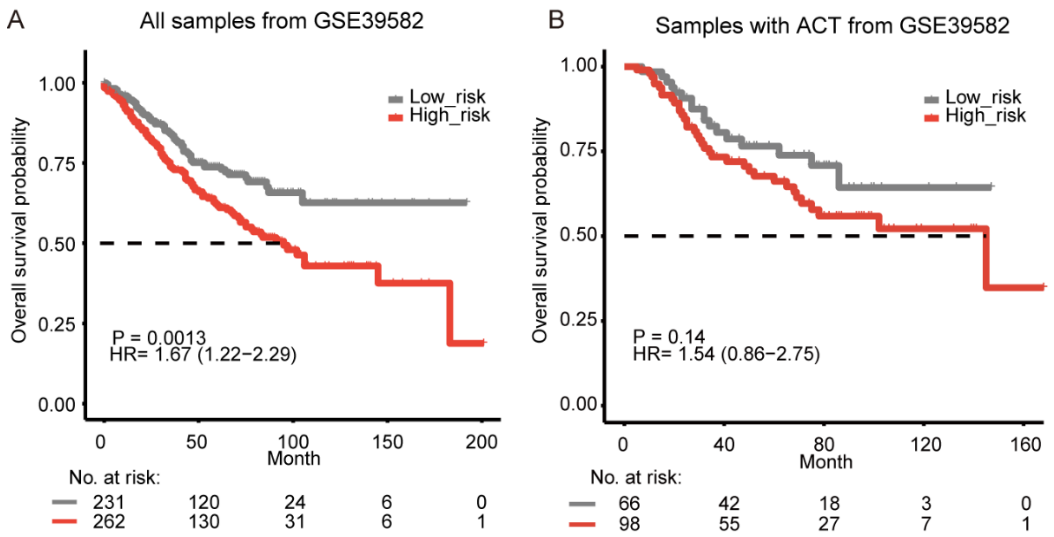

Supplementary Figure 6. Kaplan-Meier estimates of the overall survival of patients using the five gene signature in GSE39582. (A) All samples with and without fluorouracil-based adjuvant chemotherapy (ACT). (B) Samples with ACT.
